# Reviews and Book Notices

**Published:** 1882-05

**Authors:** 


### Reviews and Book Notices.

ARTICLE XIX.—THE APPLIED ANATOMY OF THE NERVOUS SYSTEM; being a Study of this Portion of the Human Body from a Standpoint of its General Interest and Practical Utility, designed for use as a text-book and a work of reference, by AMBROSE L. RANNEY, A.M., M.D. With numerous illustrations. New York: D. Appleton & Co. 1881.

The author has evidently garnered the best points of other observers in this line. His research has been extensive, and his application of anatomical facts to account for clinical results is plausible. However, the field he occupies is still debatable land, and until we have a more definite knowledge of the anatomy and physiology of the brain and nervous system, such books as this must be more speculative than authoritative.

Dr. Ranney discusses in turn the anatomy of the brain, cerebrum, corpus strictum and optic thalamus, corpora quadrigermina, cerebellum and medulla oblongata. The surgical bearings of cerebral topography are given, followed by the clinical subdivisions of the brain, physiology and practical clinical deductions.

Part II discusses the cranial nerves; part III the spinal cord, part IV the spinal nerves. Parts II and III contain many suggestions of value, although the author often has unfortunately obscured his meaning by expressing his thoughts in sentences of complex and faulty construction.

The mechanical part of the book is very good, and on the whole, it is a valuable addition to the ever increasing list of medical works. J. S. R.

